# Keeping Things under Control at the Neuromuscular Junction

**DOI:** 10.1371/journal.pbio.0040289

**Published:** 2006-07-25

**Authors:** Mary Hoff

In vertebrates, neurons signal muscles to contract by releasing a neurotransmitter, acetylcholine, into the intercellular space. They do so by fusing spherical acetylcholine-filled packages with the plasma membrane. But what controls when—and how many—such packages, known as synaptic vesicles, release their contents? A protein called tomosyn is thought to play a key role in keeping the process under control. But until now its mode of action was unknown.

To clarify tomosyn’s role in regulating acetylcholine release at the neuromuscular junction, Elena O. Gracheva, Janet E. Richmond, and colleagues enlisted the help of *Caenorhabditis elegans*, a salt-grain-sized nematode that, because of its simple nervous system and tractable genetics, is widely used to study how neurons work. *C. elegans* has a gene, *tom-1*, that makes a class of proteins, TOM-1, similar to vertebrate tomosyn. After discovering that *C. elegans* TOM-1 is biochemically very similar to tomosyn in vertebrates, the researchers decided to use the nematode as a model for exploring vertebrate tomosyn function.

They began by measuring traits of the nerve-to-muscle signal in *tom-1* mutants that are unable to properly produce TOM-1. They found that the evoked release of acetylcholine onto the muscle was enhanced in *tom-1* mutants relative to non-mutant (wild-type) organisms, due to a prolonged evoked response. Other studies have shown that tomosyn is able to block the formation of a protein complex called the SNARE complex, known to mediate vesicle release. Could this be how tomosyn regulates synaptic transmission? Before they could conclude so, the researchers had some alternative explanations to discount. By comparing post-synaptic electrophysiological activity in wild-type and mutant strains, they showed that the effects observed in the *tom-1* mutants were not due to changes in post-synaptic receptor kinetics. By comparing the morphology of neurons in wild-type and mutant strains, they also showed the effects were not due to altered neuronal connectivity. And by comparing the number and distribution of neuromuscular synapses in the two (using a technique called immunolabeling), they demonstrated the moderating effect of tomosyn is not due to changes in how synapses develop, either.

Next they tested whether the *tom-1* mutant trait could be reversed by selectively expressing TOM-1 in cholinergic neurons (acetylcholine-producing neurons). They found the nerve-to-muscle signal to be less prominent in this situation than in either wild-type or *tom-1* strains, while the duration of the response evoked in the muscle was like that of wild-type worms. The conclusion: TOM-1 regulates synaptic transmission on the nerve side, rather than on the muscle side, of the synapse.

With the hypothesis that tomosyn acts on the vesicle release part of signal transmission strengthened by these findings, the researchers decided to look more closely at the vesicles themselves. Electron microscopic examination of synapses in wild-type and mutant strains showed the number of vesicles did not differ but that vesicle distribution did, with *tom-1* mutants having more vesicles in contact with the plasma membrane and a broader distribution along the membrane. In addition, the mutant worms whose cholinergic function had been restored through selective TOM-1 expression had fewer contacting vesicles and less distributed vesicles in their cholinergic synapses.

An important part of the vesicle-signaling process at the neuromuscular junction is the preparation of vesicles for release, a process known as “priming.” Previous research has shown that synapses in a *C. elegans* mutant known as *unc-13(s69)*, which has priming problems, have few vesicles in contact with the plasma membrane and do not release acetylcholine. This suggests that the vesicles lined up on the membrane in the *tom-1* mutants represent a superabundance of primed vesicles. To test this hypothesis, the researchers produced *tom-1 unc-13(s69)* double mutants. As they predicted, these double mutants had more contacting vesicles than *unc-13(s69)* alone and restored acetylcholine release. The researchers concluded that TOM-1’s role in moderating signal transmission at the neuromuscular junction occurs by limiting the number of vesicles primed to release acetylcholine into the intercellular space.

## 

**Figure:**
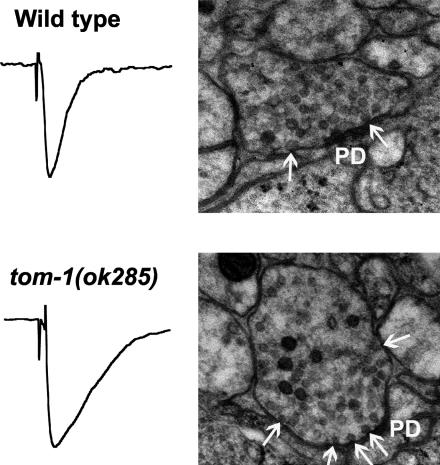
Electron micrographs of rapidly fixed worm synapses reveal differences in synaptic vesicle localization in tomosyn mutants versus wild type.

